# Differences between Outdoor and Indoor Sound Levels for Open, Tilted, and Closed Windows

**DOI:** 10.3390/ijerph15010149

**Published:** 2018-01-18

**Authors:** Barbara Locher, André Piquerez, Manuel Habermacher, Martina Ragettli, Martin Röösli, Mark Brink, Christian Cajochen, Danielle Vienneau, Maria Foraster, Uwe Müller, Jean Marc Wunderli

**Affiliations:** 1 Empa, Laboratory for Acoustics/Noise Control, Swiss Federal Laboratories for Materials Science and Technology, 8600 Dubendorf, Switzerland; 2 Swiss Tropical and Public Health Institute, 4051 Basel, Switzerland; andipiquerez@hotmail.com (A.P.); martina.ragettli@unibas.ch (M.R.); martin.roosli@unibas.ch (M.R.); danielle.vienneau@unibas.ch (D.V.); maria.foraster@unibas.ch (M.F.); 3 n-Sphere AG, 8045 Zürich, Switzerland; manuel.habermacher@n-sphere.ch; 4 Centre for Chronobiology, University of Basel, 4001 Basel, Switzerland; 5 Federal Office for the Environment, 3003 Bern, Switzerland; mark.brink@bafu.admin.ch; 6 Centre for Chronobiology, Psychiatric Hospital of the University of Basel, 4056 Basel, Switzerland; christian.cajochen@upkbs.ch; 7 Deutsches Zentrum für Luft- und Raumfahrt e.V. (DLR), Institut für Luft- und Raumfahrtmedizin, 51147 Köln, Germany; Uwe.Mueller@dlr.de

**Keywords:** sound level differences indoors/outdoors, correction factors, open window, tilted window, closed window, linear model

## Abstract

Noise exposure prediction models for health effect studies normally estimate free field exposure levels outside. However, to assess the noise exposure inside dwellings, an estimate of indoor sound levels is necessary. To date, little field data is available about the difference between indoor and outdoor noise levels and factors affecting the damping of outside noise. This is a major cause of uncertainty in indoor noise exposure prediction and may lead to exposure misclassification in health assessments. This study aims to determine sound level differences between the indoors and the outdoors for different window positions and how this sound damping is related to building characteristics. For this purpose, measurements were carried out at home in a sample of 102 Swiss residents exposed to road traffic noise. Sound pressure level recordings were performed outdoors and indoors, in the living room and in the bedroom. Three scenarios—of open, tilted, and closed windows—were recorded for three minutes each. For each situation, data on additional parameters such as the orientation towards the source, floor, and room, as well as sound insulation characteristics were collected. On that basis, linear regression models were established. The median outdoor–indoor sound level differences were of 10 dB(A) for open, 16 dB(A) for tilted, and 28 dB(A) for closed windows. For open and tilted windows, the most relevant parameters affecting the outdoor–indoor differences were the position of the window, the type and volume of the room, and the age of the building. For closed windows, the relevant parameters were the sound level outside, the material of the window frame, the existence of window gaskets, and the number of windows.

## 1. Introduction

Noise exposure prediction models that are used in health effect studies normally yield free field exposure levels outside residences as results. In these models, the sound insulation of buildings is neglected. However, to assess the sound exposure of the inhabitants inside buildings, an estimate of the indoor sound level is necessary, as people spend a considerable amount of their time indoors. Indoor sound levels are especially important at night, as they account for awakening and other sleep disturbances caused by outside noise, which are assumed to play a major role in noise-induced health impairments [1,2,3,4,5]. However, little data is available on the difference between indoor and outdoor noise levels. Most health studies therefore rely on outdoor levels [6,7]. Others apply a constant difference in terms of sound levels outdoors and indoors. The latter approach however is a very coarse estimate and does not take into account specific conditions of the dwelling situation, window opening behaviour, and building characteristics. This is a major cause of uncertainty in the prediction of the “true” noise exposure and can produce exposure misclassification in studies of noise-induced health effects, particularly during the night-time. Amundsen et al. showed that changes in sound insulation have a significant effect not only on sleep disturbance but also on annoyance ratings [8,9]. In order to overcome these limitations, Foraster et al. for example introduced different correction factors to estimate the indoor noise exposure from outdoor noise, which however needs further validation [10]. Hence, better knowledge of the noise reduction by buildings would be useful for future epidemiological research in order to reduce exposure misclassification. If the extent of exposure misclassification is independent of health status and is thus non-differential, health effects are most likely underestimated. If, however, exposure misclassification depends on health status, bias in any direction is possible, depending on the direction of the misclassification. A common coping strategy for example is to close the windows [11,12,13]. Hence, a plausible assumption for such differential exposure misclassification is the possibility that noise-sensitive individuals who are more vulnerable to health effects are likely to more often close the windows in their dwellings. As a consequence, their noise exposure is more strongly overestimated than for people with open windows. This again, would result in an underestimation of the true exposure–response association [14].

So far, only a few studies have assessed façade sound insulation compared to outdoor noise levels for open or tilted window positions. In a field study performed in Australia, an average level difference for open windows of 11 dB(A) resulted [15]. Two studies performed at the German DLR-Institut für Luft- und Raumfahrtmedizin (The German Aerospace Center (DLR) is the national aeronautics and space research centre of Germany) [16,17,18] reported similar values, ranging from 10 to 13 dB(A) depending of the type of noise source. In the same two studies, tilted, i.e., slightly open windows were also tested. While the measurements of 2006 resulted in differences of 14 and 15 dB(A) for road and air traffic, respectively, the follow-up study yielded average values of 18 to 19 dB(A) for road and railway traffic. For aircraft noise, Jansen et al. [19] as well as the Swiss Federal Office for the Environment (FOEN) [20] recommend assuming a level difference between the outdoors and indoors in the case of tilted windows of 15 dB(A). For the same noise source, Maschke et al. [21] derived a mean difference in level of 12 dB(A) for tilted window positions based on loudspeaker experiments. The European Environment Agency summarizes these results in its “Guidelines for noise exposure assessment” [22] where an attenuation of 5–10 dB(A) is recommended for open windows and 10–15 dB(A) for slightly open windows.

For closed windows, numerous building acoustic studies are at hand, showing the major influence of the façade type on the resulting sound insulation (see for example [22,23]). In addition to the room size, the reverberation time as well as window area play an important role [24,25,26]. Licitra et al. [27] performed an extensive measurement study on railway noise in Pisa, with measurements inside and outside of buildings. Pabst [28] gives differences for closed windows ranging from 24 to 35 dB(A), for aircraft noise. This study also showed that the same window can have a different sound level reduction depending on the aircraft type (up to ±3 dB(A)). The previously mentioned studies by DLR [16,17] yielded mean sound insulations in the case of closed windows ranging from 26 to 37 dB(A) depending on the sound source and the façade type. Scamoni et al. [29] analyzed a dataset of 334 locations, resulting in a mean outdoors–indoors difference for closed windows of 31 dB(A) and values ranging from a minimal difference of 18 to a maximal difference of 42 dB(A). As a conservative estimate, the FOEN [20] recommends assuming an outside–inside level difference of 25 dB(A), in order to get an estimate of the probability of awakening as a reaction. The influence of local building standards on the resulting sound insulation can be studied when comparing buildings, which have been erected for a specific purpose. While only limited information is available for apartments, there are various studies that investigated the acoustic properties of schools [30,31,32,33,34,35] indicating variations in sound insulation of more than 10 dB(A).

As a consequence, it can be stated that for open and tilted windows, only limited data is available and that for closed windows, the necessary information for an accurate prediction of the sound insulation in a specific situation is typically not at hand in the case of health effect studies with numerous participants. This is a limitation for health risk research as noise level at the ear of the inhabitant is considered most relevant from a biological point of view. Thus, better knowledge of building damping would enable a more accurate prediction of indoor noise exposure from outside values. Therefore, the aim of the present study is to determine representative differences between the sound level outdoors and indoors for open, tilted, and closed windows for buildings in Switzerland based on measurements (see also [36]). The most relevant parameters for the outdoor–indoor differences shall be determined and a statistical model shall be developed to predict the sound level difference as a function of dwelling and exposure characteristics, which may then later be applicable for refined epidemiological analyses. This study has been performed as a follow-up project of the nationwide assessment of road, railway, and aircraft noise exposure conducted within the Short and long-term effects of Transportation Noise Exposure (SiRENE) study [6,7,37,38].

## 2. Methods

### 2.1. Measurements

From the 5592 respondents of the socio-acoustic survey of SiRENE [38], 102 participants that agreed to be contacted again were visited at home. Interviews on noise annoyance, coping strategies, and sound level measurements were carried out by three research assistants between May and November 2016. The analysis of these interviews however is not part of this paper. Inclusion criteria were that the participants lived nearby heavily used roads, and the $L_{den}$ at the highest exposed façade had to be ≥50 dB(A) to ensure sufficient outdoor noise to be detected inside. Measurements were carried out in apartments at different floor levels. Over 80% of the measurements were performed at ground floor up to the third floor. The remaining 20% of measurements were taken between the fourth and seventh floors.

The sound recordings were performed simultaneously outdoors, with the microphone flush mounted in the middle of the outer face of a window, and indoors, if possible in the bedroom at the position of the pillow (hypothetical position of sleeper’s ear). In the case the sleeping room was not the room most exposed to noise, the measurements were repeated in the living room (N = 55) with the microphone placed in the middle of the room at a height of approximately 1.5 m. Therefore, three scenarios with open, tilted and closed windows were recorded. As the aim of this study was to determine sound level differences between the indoors and outdoors, but not to establish a representative long-term sound exposure, it was decided to take short, but fully controlled measurements in order to minimize the impact of indoor noise sources on the measurements. Each scenario was therefore measured over three minutes. During the measurements, great attention was given to minimize any sounds originating from inside the building. If there were interfering noises inside or there were unwanted sound sources other than road traffic noise outside, the measurements were stopped and repeated. Measurements were not taken at a specific time of day, but according to the availability of the participants, they were typically taken in the evening hours. Altogether, measurements were carried out in 157 rooms in 102 buildings. About 80% of these were in flats in apartment buildings and 20% were single-family houses.

For the outdoor measurements, class II Noise Sentry RT (Convergence Instruments, Sherbrooke, QC, Canada) sound level meters were used, which logged A-weighted 1 s $L_{eq}$ levels. This device has a dynamic measurement range of 31 to 117 dB. This class II sound level meter was chosen because these devices were used in the same time for long-term measurement over one week to validate the noise exposure modeling of SiRENE [39]. As the outdoor microphone was flush-mounted a frequency-independent pressure doubling can be assumed. Therefore, a correction of −6 dB was applied to get free field conditions. The indoor measurements were performed with the class I sound level meter, type NTI XL2 (NTi Audio AG, Schaan, Liechtenstein) with a free field microphone. This device has a dynamic measurement range of 17 to 138 dB. One-third octave-band spectra from 50 Hz to 10 kHz were recorded indoors with a temporal resolution of one second (1 s $L_{eq}$). Both sound level meters were calibrated before each measurement.

**Figure 1:**
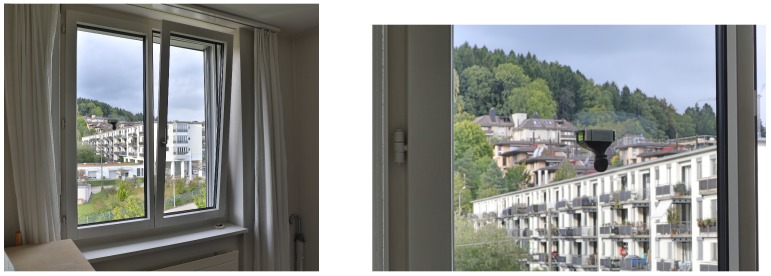
(**Left**) An example of a typical window with two sashes in a tilted position. The Noise Sentry is mounted on the left side, in the middle of the window pane; (**Right**) Close-up showing the Noise Sentry mounted on the window.

For each recording situation, additional parameters describing the room and sound insulation properties were collected using a pre-defined protocol. These parameters were chosen as possible predictors based on consultation of experts in building acoustics and our own experience. Table 1 shows a complete list of the parameters. Figure 1 shows a typical window with two sashes (the moveable part of the window). Overall, 87% of the analyzed windows had two sashes and only 13% had only one sash. The opening angle of tilted windows was typically about 5–10 degrees.

### 2.2. Calculation of the Sound Level Differences between the Outdoors and Indoors

**Figure 2:**
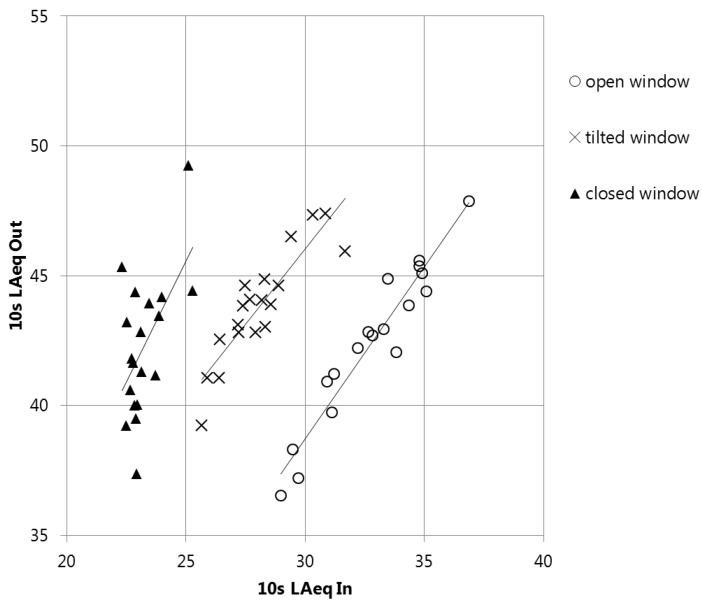
Example of measurement data (ID 0342): 10 s $L_{Aeq}$ outdoors (corrected by −6 dB) versus indoors. In this case the median outdoor–indoor difference for the open (circles, $R^{2}=0.92$) and tilted (crosses, $R^{2}=0.79$) windows with $R^{2}\geq 0.45$ are included in the further analysis. As there is no clear outdoor–indoor correlation for the closed window (triangles, $R^{2}=0.32$), this measurement is not considered in the statistical analyses.

To estimate the mean difference between the sound pressure level outdoors and indoors, the following procedure was carried out. For a measurement period of approximately three minutes per room and window scenario, an energetically mean sound level was calculated for intervals of 10 s. This was primarily done in order to account for a possible slight time offset due to inexact synchronization between the devices (±2 s). For each 10 s $L_{Aeq}$, the difference between the outdoors and indoors was calculated. These approximately 18 data points (in 3 min) were plotted, a linear fit was applied, and the correlation between the sound levels outdoors and indoors was evaluated (Figure 2 shows an example). All situations with an $R^{2}\geq 0.45$ were classified as potentially valid measurements. As a second criterion, the slope (dB/dB) had to be close to 1. For measurements with a slope <0.5 or >1.5 it was visually verified whether a plausible correlation between the indoors and outdoors existed. This leads to the general restriction that more often in measurements with closed windows, windows that have a high sound insulation and places with low sound levels outdoors are not considered in the statistical analyses. From the $\Delta 10\;s\;L_{Aeq}\left(out-in\right)$ the median was taken as representative difference for the specific situation.

### 2.3. Statistical Analyses

The statistical analyses were carried out with R version 3.1.3. In a first step, a boxplot for each window position was plotted and analyzed. Outliers were removed following Tukey’s method [40]. Therefore, outliers were defined as outside 1.5 times the interquartile range (IQR). The method was applied on each window position separately.

A multiple linear regression analysis was used to model the influence of the predictors listed in Table 1 on the outdoor–indoor sound level difference. These models combine categorical variables, continuous variables, and interactions to predict the dependent variable ($\Delta L_{Aeq}out-in$). The parameters volume of the room and age of the building were only available in categories. Therefore, these two actually continuous parameters were considered as categorical. The assumption that the errors are normally distributed was tested and a linear model (lm in R) was applied.

The process of sound transmission is fundamentally different in the case of closed windows. With open and tilted windows, the opening is the dominant sound path and material properties can in most cases be neglected. With windows closed, properties like the composition of the multi-layer glazing, the material of the window frame, window gaskets, etc. are important. This results in many potential influencing parameters and interactions. Therefore, separate regression models were established for closed windows on the one hand and for open and tilted windows on the other hand. The variable selection was done by a stepwise approach with the Akaike Information Criteria (AIC), where the model with the lowest AIC was preferred (function step in R). Variables were retained if statistically significant (*p*-value ≤ 0.05). Compliance with the model assumptions was confirmed by visual inspection of the residual plots (Tukey–Anscombe plot, normal plot, and scale-location plot). Possible leverage or influential data points were detected by inspection of the leverage plot.

## 3. Results

### 3.1. A-Weighted Sound Level Differences between Outdoors and Indoors

**Figure 3:**
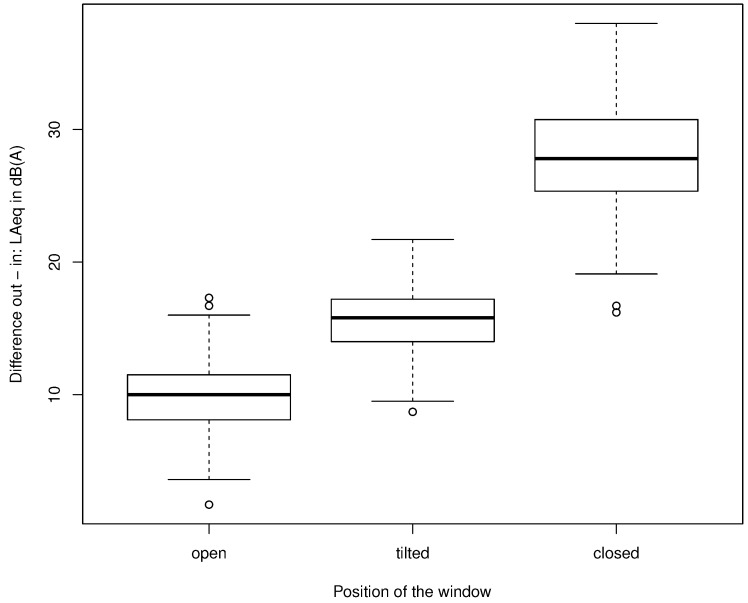
Boxplots of all valid data showing the median (horizontal line in boxes), the 25% and 75% quantiles (lower and upper boundaries of boxes), the whiskers comprising the data within 1.5 times the interquartile range, and outliers outside the whiskers.

From the measurements in 157 rooms, 115 measurements for open windows, 116 measurements for tilted windows, and 76 measurements for closed windows were valid. Results for the outdoor–indoor differences are shown in Figure 3, with corresponding values given in Table 2. In brief, the median ± standard deviation was 10.0 ± 2.9 dB(A), 15.8 ± 2.7 dB(A), and 27.8 ± 4.4 dB(A) for open, tilted, and closed windows, respectively. For the statistical analyses, the six outliers in the boxplots, defined as outside 1.5 times the interquartile range, were removed.

### 3.2. Spectral Sound Level Differences between the Outdoors and Indoors

**Figure 4:**
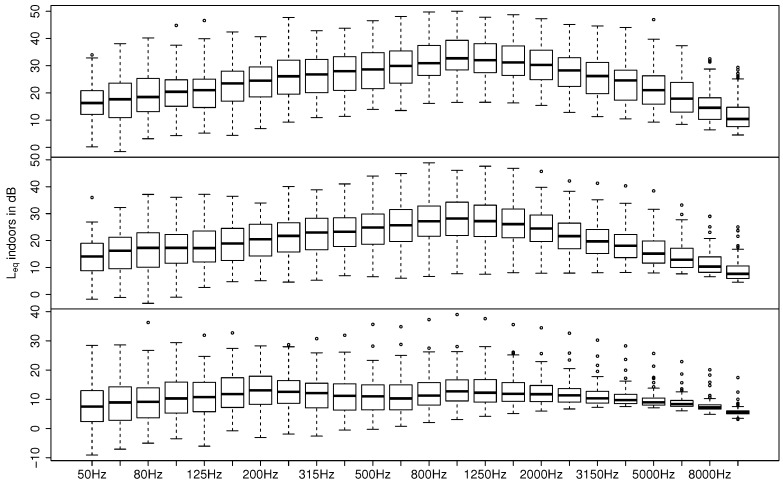
$L_{eq}$ indoors in one-third octave bands for open (top), tilted (middle), and closed windows (bottom) of all valid measurements. Boxplots show the median (horizontal line in boxes), 25% and 75% quantiles (lower and upper boundaries of boxes), whiskers comprising the data within 1.5 times the interquartile range, and outliers outside the whiskers show each one-third octave band.

Figure 4 shows a statistical representation of the measured $L_{eq}$ indoors in one-third octave bands from 50 to 10,000 Hz for open, tilted, and closed windows. Both the open and tilted situations show maxima around 1 kHz, with a slight decrease of levels towards lower frequencies and a more prominent decrease of levels towards higher frequencies. In contrast, for closed windows a widely flat spectrum is shown.

**Figure 5:**
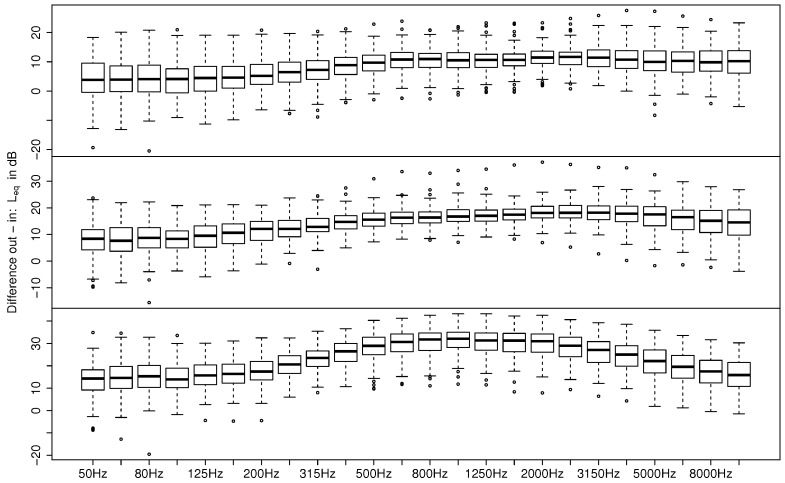
Outdoor–indoor differences for the open (top), tilted (middle), and closed windows (bottom) for all valid measurements. Boxplots show the median (horizontal line in boxes), 25% and 75% quantiles (lower and upper boundaries of boxes), whiskers comprising the data within 1.5 times the interquartile range, and outliers outside the whiskers for each one-third octave band.

As mentioned in Section 2.1 only A-weighted levels are available for the outside measurement position. In order to still get a frequency-dependent difference in level between the outdoors and indoors, an estimation of the spectra outdoors was done. For that purpose, we assumed a typical road noise spectrum in a distance <200 m with 5% heavy vehicles and a velocity of 50 km/h based on the CNOSSOS road traffic model [41]. The resulting spectral outdoor–indoor differences for open, tilted, and closed windows are shown in Figure 5. In the case of open and tilted windows, we see more or less flat curves with a frequency-independent attenuation, which is in good agreement with the literature [18,42]. For closed windows, in contrast the noise reduction seems to be higher for frequencies ranging from 400 to 4000 Hz. While a smaller damping at lower frequencies can be expected for situations with closed windows (see for example [23,43], a decay towards higher frequencies seems to be counter-intuitive. This finding however can be explained by the residual sound of the measuring device in combination with the very low sound pressure level. As can be seen in Figure 4 the levels drop below 10 dB at very low as well as very high frequencies. The sound level meter type NTI XL2 on the other hand exhibits a residual noise of more than 10 dB at the one-third octave band of 63 Hz and below, and a residual noise of more than 6 dB at one-third octave bands of 4 kHz and higher. Consequently, it can be concluded that at these frequencies residual noise influenced the measurement and that the real sound insulation is therefore underestimated. Even though the mentioned frequency range is not dominant with respect to A-weighted levels, a slight underestimation of the resulting level difference between outside and inside must be assumed for the situation of closed windows as given in Figure 3 and Table 2.

### 3.3. Linear Regression Model for Open and Tilted Windows

Based on the available predictor variables in Table 1, the following linear model was found to be appropriate:

(1) $$\Delta L_{Aeq}out-in=\beta_{0}+window+room+V+age\;\left[\mathrm{dB}\left(A\right)\right]$$

In this equation, the dependent variable is the level difference $\Delta L_{Aeq}out-in$, $\beta_{0}$ is the overall mean of this difference, and window position (open or tilted), room type, volume (V), and age are fixed categorical factors. These parameters have a significant effect with the following *p*-values: the window position, i.e., open or tilted with p<0.001, the type of room with p<0.001, the age of the building with p=0.001, and the volume of the room with p=0.01 (see Table 3). No significant interactions between the different significant parameters were detected.

This linear model yields an explained variance of 65% (adjusted $R^{2}$). The position of the window accounted for 58% of the variability in the data. Another 7% of the variance was explained by the room type (5%) and the age of the building (2%). The volume of the room does not explain much of the variability (1%).

By visual inspection of the residual plots, the compliance with the model assumptions could be confirmed. The normal distribution of the residuals was also confirmed by the Lilliefors (Kolmogorov–Smirnov) normality test (p=0.744). No leverage or influential data point was detected.

A small influence on the outdoor–indoor differences however is attributed to the mean sound level outdoors (p<0.001, explaining 3% of the variance). This critical finding is discussed in Section 4.2. The original category 35–60 m${}^{3}$ did not show a significant difference to the smaller rooms (15–35 m${}^{3}$) in the parameter estimate. Therefore, we reclassified the room volume into only two categories, namely <60 m${}^{3}$ and 60–150 m${}^{3}$.

### 3.4. Linear Regression Model for Closed Windows

**Figure 6:**
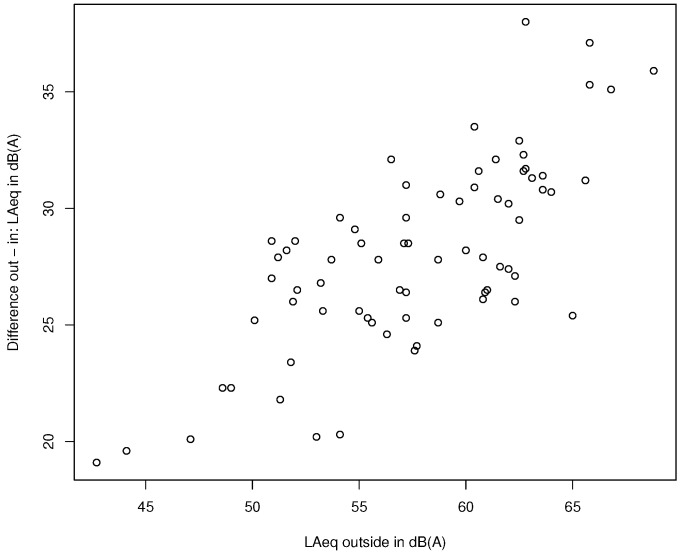
Plot showing the outdoor–indoor differences for measurements with closed windows as a function of the energetically averaged sound level outdoors. Note: In Section 2.1 it was stated that only situations with $L_{den}$ levels ≥ 50 dB(A) were selected. However, the plot also shows considerably lower levels. This alleged contradiction is due to the fact that measurements were not only performed at the most exposed façades but also at the averted façades. In addition, short-term Leq cannot directly be compared with $L_{den}$, as the latter represent long-term averages and also include penalizations.

For closed windows the main influence on the indoor–outdoor differences is attributed to the mean sound level outdoors with an $R^{2}$ of 0.55 (see Figure 6). This critical finding is discussed in detail in Section 4.3.

If we include the sound level outdoors as a parameter, despite the mentioned critical finding, the following linear model for closed windows is derived:

(2) $$\Delta L_{Aeq}out-in=\beta_{0}+\beta_{1}\cdot windows+\beta_{2}\cdot L_{Aeq}out+frame+gaskets\;\left[\mathrm{dB}\left(A\right)\right]$$

In this equation, the dependent variable is $\Delta L_{Aeq}out-in$, $\beta_{0}$ is the intercept, the material of the window frame (frame) and the existence of window gaskets (gaskets) are fixed factors, and the number of windows (windows) and the $L_{Aeq}$ outdoors are the covariates with the corresponding regression coefficients $\beta_{1}$ and $\beta_{2}$. The number of windows (p=0.02), the sound level outdoors (p≤0.001), the material of the window frame (p=0.001) and the existence of window gaskets (p=0.05) significantly influence the outdoor–indoor difference. No significant interactions between the different parameters were detected. The model coefficients are presented in Table 4. This linear model yields an explained variance of 62% (adjusted $R^{2}$).

By visual inspection of the residual plots, the compliance with the model assumptions could be confirmed. The normal distribution of the residuals was also confirmed by the Lilliefors (Kolmogorov–Smirnov) normality test (p=0.263). One measurement with a room volume smaller than 15 m${}^{3}$ was excluded, as this was the only measurement in such a small room and generated a leverage of one. After excluding this data point, no other leverage or influential data point was present in the leverage plot. The category metal window frames did not show a significant difference to wooden window frames in the parameter estimate. Therefore we put synthetic and metal window frames together in one category, representing newer windows as compared to wooden window frames.

## 4. Discussion

### 4.1. Sound Level Differences between the Outdoors and Indoors

In this study, over 300 measurements were carried out at people’s homes in a sample of more than 100 Swiss residents. The median differences between sound levels outdoors and indoors were 10.0±2.9 dB(A) for open, 15.8±2.7 dB(A) for tilted, and 27.8±4.4 dB(A) for closed windows. The ranges from the minimal to the maximal values were 16, 13, and 22 dB(A) for open, tilted, and closed windows, respectively. In case of closed windows, the sound insulation depended very much on building properties, especially the windows (glazing, material of window frame, window gaskets, etc.). This means that for a specific situation the real difference can deviate significantly from the median difference between the sound level outdoors and indoors, as measured in our sample.

In Table 5, these results are compared with the studies already mentioned in the introduction. Most studies [15,16,18,29] used a measurement position outdoors 1 to 2 m from the front of the façade. In order to correct for reflections from the building façade, Ryan et al. [15] subtracted 2.5 dB(A) to get free field conditions. In the DLR-studies [16,18] a correction of −3.0 dB(A) was applied. Hence, Ryan et al. assumed a prominent reflection from the building with a slightly lower intensity than direct sound, while Müller et al. assumed a doubling of the sound intensity by the building reflection. The results published by Scamoni et al. [29] originally did not include a correction to free field levels. Therefore, in order to get a better comparability 3.0 dB(A) were subtracted for the representation in Table 5.

- A comparison of the values for **open window situations** shows in general a good agreement between the different studies, with a range of levels from 10 to 13 dB(A). The deviation between the resulting averages is rather small, when comparing it with the substantial spread of the individual values within the studies.
- For **tilted windows** the resulting outside–inside differences are slightly greater, ranging from 14 to 19 dB(A). However, the results between the studies still look consistent. The additional sound insulation effect of a tilted window compared to an opened one can consequently be deduced as 4–6 dB(A) on average.
- For **closed window situations**, the resulting averages between the different studies are rather close, ranging from 26 to 31 dB(A). Hence, the effect of closing windows, compared to an open window situation, results on average in a level decrease inside the building of 16–18 dB(A).

Even though the different studies yielded comparable averages in the case of closed windows also, it has to be mentioned that the individual spread of measured differences within the studies was much larger than for open or tilted situations. This of course reflects the strong influence of the sound insulation properties of the different window and façade types. Some studies [15,18] also suggested an influence of the window and façade type for situations with open and tilted windows. To our understanding, such findings are rather caused by measurement uncertainties, the small number of samples, and other influencing parameters. The sound insulation of façade elements as well as window glazings is generally significantly greater than the measured level differences according to Table 5. This confirms the assumption that the dominating sound path is in both cases through the opening. The sound insulation spectra depicted in Figure 5 with a rather frequency-independent level reduction also indicate that the source spectrum cannot be a major source of influence. Other factors however are likely to play a significant role and are responsible for the differences between studies and reported situations. To our understanding, the following three aspects are most important:

- We are convinced that the angle of sound incidence, in combination with the orientation of the opening of the window, is primarily responsible for the stated source-specific effects.
- The window size and the opening angle define the opening area available for the sound passage and hence the incoming sound intensity.
- The level inside is not only defined by the incoming sound intensity but also by the room acoustic conditions in the receiving room, primarily the reverberation time and the room size.

In the DLR studies, all measurements were done in sleeping rooms with rather small windows in comparison for example to our study. Other studies (for example [15,29]) included other room types like empty rooms, bathrooms, offices, nurseries, and schools with a wide variety of room characteristics. Last but not least, the measured quantity can also have an influence on the resulting sound level differences. This effect however is assumed to be rather small. The DLR study of 2006 [16] identified an average difference between maximum sound pressure levels $SPL_{ASmax}$ and equivalent continuous sound pressure level $L_{Aeq}$ of ≤0.5 dB(A).

The recommendations of the European Environment Agency [22] and the FOEN [20] are in good agreement with the study results for tilted windows. However, for open window situations the range of 5–10 dB(A) given by [22] seems rather low. Also, the proposed difference in level for closed windows of 25 dB(A) proposed by the FOEN [20] seems rather conservative. While such a cautious choice might be appropriate for noise legislation purposes, it is not advisable for epidemiological studies as it introduces a systematic bias.

### 4.2. Linear Regression Model for Open and Tilted Windows

For open and tilted windows, apart from the position of the window, the room type and the age of the building turned out to be highly significant parameters. In living rooms the outdoor–indoor differences were slightly lower (−1 dB) and in kitchen/dining rooms clearly lower (−5 dB) than in bedrooms. This can be explained by more absorbing materials in bedrooms (bed, curtains, carpets), which is normally also true for living rooms (sofa, curtains, carpets). Kitchen/dining rooms generally have more sound-reflecting surfaces. Bigger rooms with the same incoming sound power have a lower sound level inside and therefore a larger indoor–outdoor difference, which the statistical model shows for rooms with 60–150 m${}^{3}$ (+1.1 dB). The volumes of living rooms and kitchen/dining rooms are generally bigger than those of sleeping rooms. The age of the building effect shows the counter-intuitive trend that newer buildings have slightly lower outdoor–indoor differences (20–40 years: +1.7 dB, >40 years: +1.9 dB). It is expected that newer buildings have greater sound insulation. Nevertheless, the effect of the age can be explained: it might reflect the bigger window sizes of newer buildings. Unfortunately, the window size was not measured. For future studies, it is suggested to collect this important parameter. The distance from the microphone inside to the window does not significantly affect the sound level differences between the inside and outside, which supports the assumption of a diffuse sound field.

A small influence on the outdoor–indoor differences is attributed to the mean sound level outdoors. It has to be assumed that this finding indicates a limitation of the measurement concept. Despite removing data points without the outdoor–indoor correlation, there might be some measurements with disturbing noises inside that influence the median difference. This effect is bigger in case of closed windows, as described in the next section.

A sensitivity analysis based on a model with all data (including four outliers) provided similar results (same parameters and significance levels, only slightly different coefficients).

### 4.3. Linear Regression Model for Closed Windows

The statistical analysis for the closed windows dataset showed a high correlation of the outside sound pressure level on the outdoor-indoor difference. This might be explained by the fact that buildings close to noisy streets have more often windows with a high level sound insulation, especially in Switzerland where extensive noise mitigation programs have been realized in the past decade. Additionally, for the last 30 years Swiss building regulations have specified minimal sound insulation depending on the outside noise level [44]. However it must be assumed that this strong influence indicates (at least partially) a limitation of the measurement procedure, as high levels of sound insulation cannot be exactly measured with typical levels of outside traffic noise. For that purpose, alternative methods with artificial source, i.e., a loudspeaker, should be considered. In addition, it has to be kept in mind that rooms with high levels of sound insulation less likely comply with inclusion criteria in the statistical analysis due to a lack of correlation between the sound levels outdoors and indoors. Hence, it must be concluded that the dataset for closed windows is likely to be unbalanced, with a tendency to underestimate the real difference in level.

Other significant parameters were the glazing composition, the material of the window frame, the existence of window gaskets, and the number of windows, however with a rather small overall effect. The linear model indicates that vinyl and metal window frames have a higher (+1.9 dB(A)) outdoor–indoor difference compared to wooden window frames. This is not as would be expected, as new wooden windows typically exhibit a higher sound insulation due to the additional mass and other construction details. However, wooden window frames are probably more often older, as vinyl window frames are more common these days. Older window frames might often be distorted, especially in case of wooden frames, and therefore not completely airtight and less sound insulated.

Windows without window gaskets have lower (−2.4 dB(A)) outdoor–indoor differences, as would be expected due to a reduced sound insulation. The more windows there are, the lower is the outdoor–indoor difference (−0.8 dB(A) · number of windows). The façade normally has clearly higher sound insulation than windows. More windows will consequently have a negative effect on the overall sound insulation.

Sensitivity analyses based on a model with all data (including two outliers) provided slightly different results: in this case only the outside sound pressure level had a significant effect. A closer look at the two outliers shows that the outdoor–indoor difference is below 18 dB(A), although there are no parameters indicating very low sound insulation (both having gaskets, double and triple glazing, etc.). Therefore, we do not trust the two measurements with a very low outdoor–indoor difference.

## 5. Relevance and Applicability

The proposed measurement and analysis concept was proven to be suitable to determine differences between sound levels outdoors and indoors for open and tilted windows. Limitations are the short measurement time of only 3 min and the fact that these took often place in the evening hours when the participants were at home. However, the measurements were performed under observation and periods with interfering sounds were excluded. As it is not the intention of the measurement procedure to establish representative long-term sound exposure levels, such short but fully controlled measurements are expected to be advantageous compared to long-term measurements, where outside noise is mixed with often dominant interior noises. Therefore, we are convinced that the procedure yielded reliable outside–inside differences. For closed windows however, using the present road traffic as sound source instead of artificial source, turned out to be a limiting factor, causing a tendency to underestimate the real sound insulation for façades meeting higher building standards. The resulting outside-inside differences seem applicable in any case, however they should be interpreted as a safe side estimate.

All measurements for this study were performed in Switzerland. Even though the measurement sites were not specifically selected to reproduce an average Swiss building standard, with measurements in over 100 buildings we assume that the results are representative for situations with high road traffic noise exposure in Switzerland. The comparison with other studies, as shown in Table 5, reveals a certain consensus on the insulation effect of open or tilted windows. As the dominant sound path goes through the opening, window as well as façade properties are of minor influence. Other influencing factors, such as the angle of sound incidence or the size of the opening, cause a variation of results. The latter however still lies within 1–2 dB(A), a range which is comparable to other uncertainties in source and propagation modeling. Consequently it can be concluded that the results for open and tilted windows presented in this study have general validity and might be applied in other countries also. However, the situation looks different in the case of closed windows. Here, the building standard has a major influence on the outcome and the range of possible types of sound insulation is much broader. It has also to be taken into account that in many countries the requirements for façade sound insulation and quality of windows depend on the outdoor noise level. Therefore, the range of applications of the presented results should be restricted to countries with a comparable building standard to Switzerland, such as for example most countries in Northern Europe.

## 6. Conclusions

A measurement method is presented that allows to assess the sound insulation of buildings based on simultaneous measurements of traffic noise outdoors and indoors. On that basis comprehensive measurements have been performed to derive representative level differences outside to inside for open, tilted and closed windows. It could be shown that the measurement and analysis procedure yields reliable results for open and tilted windows. However in the case of situations with closed windows and high sound insulations sometimes the signal-to-noise ratio, using existing traffic noise instead of an artificial sound source, is a limiting factor.

Based on additional parameters such as the orientation towards the source, floor and room type as well as sound insulation characteristics a statistical model was established to predict outside-inside level differences. In combination with noise mappings that describe noise levels outside of buildings this model can be used to estimate the sound exposure inside of dwellings, an information which is mandatory to assess sleep disturbance and can also be applied in epidemiological or socio-acoustic studies.

## Figures and Tables

**Table 1:** Considered parameters describing the room and its sound insulation characteristics used as possible predictors for sound level differences outdoors/indoors.

Parameter	Type	Levels	No. of Levels
Window position	Categorical	Open, tilted, closed	3
Floor level	Continuos	0, 1, 2, etc.	-
Room type	Categorical	Sleeping room, living room, kitchen/dining room	3
Orientation of window towards source	Categorical	frontal, lateral ($90^{\circ }$), opposite side	3
Distance to source	Continuous	Distance in m	-
Microphone position inside	Categorical	Corner, close to wall, free in the room	3
Distance microphone inside-window	Continuous	Distance in m	-
Window-frame	Categorical	Wood, synthetic material, metal	3
Existence of window gaskets	Categorical	yes, no, unknown	3
Condition of window gaskets	Categorical	Good, mediocre, bad, unknown	4
No. of window glasses	Categorical	Single, double, triple glazing	3
Type of window	Categorical	1 sash (moveable part of the window), 2 sashes	2
Type of façade	Categorical	Façade with single windows, band of windows, glass front	3
No. of windows in room	Continuous	1, 2, 3, etc.	-
Proportion of glazed area	Continuous	Percentage, relative to the wall area	-
Volume of the room	Categorical	<15, 15–35, 35–60, 60–150 m${}^{3}$	4
Type of building	Categorical	single-family house, detached apartment building, continuous block of flats	3
Age of building	Categorical	>40, 20–40, <20 years, unknown	4
Period of renovation	Categorical	1971–1980, 1981–1990, 1991–2000, not renovated, unknown	5
Aeration	Categorical	Window ventilation, artificial ventilation	2
Room characteristics	Categorical	Corner room, top floor with pitched roof area, other	3

**Table 2:** Differences of the sound levels outdoors (corrected by –6 dB, representing free field conditions) and indoors for the different window positions and the corresponding number of measurements. (SD: standard deviation)

Window Position	$\Delta 10\;s\;L_{\mathrm{Aeq}}\left(\mathrm{out}-\mathrm{in}\right)$				Number of Measurements
	Median (25%, 75% Quantile)	Min	Max	SD	
open	10.0 (8.1, 11.5)	1.7	17.3	2.9	115
tilted	15.8 (14.0, 17.2)	8.7	21.7	2.7	116
closed	27.8 (25.4, 30.8)	16.2	38.0	4.4	76
all					307

**Table 3:** Parameter estimates of the regression model for outdoor–indoor sound level differences for open and tilted windows. (V: volume. CI: confidence interval.)

Parameter	Symbol in Equation (1)	Coeff.	95% CI	Std. Error	*t* Value	Pr (>|t|)
Intercept	$\beta_{0}$	8.5	[7.5;9.5]	0.5	16.8	<0.001
Window position	window = open	0 ${}^{a}$				
	window = tilted	6.1	[5.4; 6.7]	0.3	19.1	0.001
Room	room = bedroom	0 ${}^{a}$				
	room = kitchen/dining room	−5.1	[−7.3; −2.9]	1.1	−4.5	<0.001
	room = living room	−1.1	[−1.9; −0.4]	0.4	−3.0	0.003
Room volume	V = <60 m${}^{3}$	0 ${}^{a}$				
	V = 60–150 m${}^{3}$	1.2	[0.2; 2.1]	0.5	2.4	0.018
Age of building	age < 20 years	0 ${}^{a}$				
	age = 20–40 years	1.7	[0.7; 2.7]	0.5	3.3	0.001
	age > 40 years	1.9	[0.9; 2.9]	0.5	3.7	<0.001

0 ${}^{a}$ Reference values.

**Table 4:** Parameter estimates of the regression model for indoor–outdoor sound level differences for closed windows.

Parameter	Symbol in Equation (2)	Coeff.	95% CI	Std. Error	*t* Value	Pr (>|t|)
Intercept	$\beta_{0}$	−3.03	[−9.2; 3.2]	3.11	−1.0	0.334
Number of windows	$\beta_{1}$	−0.93	[−1.7; −0.16]	0.38	−2.4	0.018
$L_{Aeq}$ outdoors	$\beta_{2}$	0.55	[0.4; 0.7]	0.05	10.2	<0.001
Material of the window-frame	frame = wood	0 ${}^{a}$				
	frame = synthetic/metal	1.91	[0.7; 3.1]	0.59	3.2	0.002
Existence window gaskets	gaskets = yes	0 ${}^{a}$				
	gaskets = no	−2.32	[−4.6; 0.0]	1.15	−2.0	0.050

0 ${}^{a}$ Reference values.

**Table 5:** Differences in the sound levels outdoors and indoors: a comparison with other studies. The values in brackets give the number of analyzed locations. (DLR: German Aerospace Center.)

Window Position	This Study	DLR 2010 [18]			DLR 2006 [16]		Scamoni 2014 [29]	Ryan 2011 [15]	Maschke 2010 [21]	BUWAL 1998 [20]
		Freight Trains	Passenger Trains	Road	Road	Aircraft	Reference Road	Road	Aircraft	Aircraft
open	10.0 (115)	11.3 (4)	11.9 (4)	11.6 (4)	13.4 (4)	10.0 (4)		10.7 (11)		
tilted	15.8 (116)	18.6 (10)	18.0 (10)	17.7 (10)	13.7 (32)	15.3 (32)			12	15
closed	27.8 (76)	30.1 (13)	29.7 (13)	30.1 (13)	27.0 (15)	25.6 (15)	31.2 (334)			25
